# Evolutionary and functional constraints structure human gene research visibility

**DOI:** 10.1186/s12864-026-13001-5

**Published:** 2026-06-03

**Authors:** Eswarrijah Eswaran, Marvin Zimmermann, Gamchini Krishnagopalan, Jelja M. Ottersbach, Toni I. Gossmann

**Affiliations:** Computational Systems Biology, TU Dortmund University, Emil-Figge-Str. 66, 44227 Dortmund, Germany

## Abstract

**Background:**

Biomedical research effort is distributed highly unevenly across human genes, with a small subset dominating the scientific literature while thousands remain sparsely studied. Whether this imbalance reflects intrinsic biological importance or historically reinforced research bias remains unclear. Understanding how research attention relates to gene properties is essential for more systematic exploration of the human genome.

**Results:**

Here, we quantify gene-level publication patterns and integrate sequence features, evolutionary constraint, gene age, expression, and disease associations across stratified gene sets. Using standardized MANE Select annotations, we show that publication counts follow a strongly heavy-tailed distribution. Highly studied genes cluster within a narrow GC-content regime and exhibit lower nonsynonymous substitution rates and lower dN/dS ratios, consistent with stronger long-term evolutionary constraint. In contrast, genes sampled from below rank 10,000 are enriched for evolutionarily younger loci and display moderately elevated dN and dN/dS values, reduced expression magnitude, and increased tissue specificity. At the disease level, research attention concentrates within a limited number of dominant domains, particularly cancer, respiratory, and vascular diseases, whereas congenital and rare disease categories remain comparatively underrepresented. Genes associated with orphan diseases show significantly reduced publication counts.

**Conclusions:**

Together, these results demonstrate that research attention is systematically structured across evolutionary, molecular, and disease dimensions. The least-studied genes represent a distinct and underexplored portion of the genome, characterized by features that may reduce experimental tractability. These findings highlight the need for bias-aware research prioritization strategies to broaden discovery and ensure more comprehensive characterization of human genes.

**Supplementary Information:**

The online version contains supplementary material available at 10.1186/s12864-026-13001-5.

## Key points

- Research attention is highly uneven. A small fraction of genes dominates the biomedical literature, while thousands remain systematically understudied.
- Visibility correlates with evolutionary history. Highly studied genes tend to be older and more evolutionarily constrained, whereas low-visibility genes are enriched for younger and moderately less constrained loci.
- Disease context amplifies bias. Cancer and common disease domains concentrate gene-level attention, while rare and congenital disease categories remain comparatively neglected.
- Future research must be bias-aware. Incorporating evolutionary constraint, gene age, and visibility metrics into prioritization frameworks can broaden systematic discovery.

**Supplementary Information:**

The online version contains supplementary material available at 10.1186/s12864-026-13001-5.

## Introduction

The human genome encodes approximately 20,000 protein-coding genes, yet biomedical research effort is distributed strikingly unevenly across them. A small subset of loci dominates the scientific literature, accumulating thousands of publications, while thousands of genes remain sparsely characterized [1, 2]. Whether this imbalance reflects intrinsic biological importance or instead arises from historically reinforced patterns of investigation remains an open question. Understanding the structure of research attention is essential, as it shapes hypothesis generation, funding allocation, model-system development, and ultimately the trajectory of biomedical discovery.

Multiple sociotechnical factors contribute to this skew. Genes implicated in common diseases, embedded in densely connected molecular networks, or supported by extensive reagent availability tend to attract sustained investigation [3, 4]. Academic incentives further reinforce incremental advances on well-established systems [5–7]. Once a gene becomes experimentally tractable and richly annotated, it becomes easier to reuse, validate, and extend, generating a cumulative advantage that amplifies visibility over time. Conversely, genes lacking initial tools, clear phenotypes, or disease associations may remain persistently underexplored.

However, research attention may not be driven solely by sociological inertia. Intrinsic biological properties shape experimental tractability. Genes that are deeply conserved across evolutionary time, under strong purifying selection, and broadly expressed across tissues are more likely to possess stable orthologs in model organisms and to yield reproducible phenotypes across systems. In contrast, evolutionarily younger genes, lineage-specific innovations, or genes with highly tissue-restricted expression may require specialized physiological contexts or human-relevant experimental systems, posing practical challenges for standard model-organism pipelines. Such differences suggest that the landscape of research visibility may partially reflect underlying evolutionary and functional constraints.

Evolutionary theory provides a framework for quantifying these properties. Long-term selective constraint, often approximated by nonsynonymous substitution rates (dN) or dN/dS ratios, captures the degree to which protein sequences tolerate change [8]. Gene age, inferred through phylostratigraphy, reflects the evolutionary depth of a locus and correlates with network integration and functional breadth. Expression magnitude and tissue specificity further inform whether a gene operates as a broadly required cellular component or within specialized biological contexts. Although these attributes are known to covary [9–12], their relationship to research prioritization has not been systematically characterized at genome scale.

Beyond individual genes, research attention is also structured at the level of disease domains. Certain areas, particularly cancer and other common complex diseases, concentrate substantial molecular investigation, whereas rare, congenital, metabolic, or tissue-specific disorders often receive comparatively limited attention [4]. Whether such disparities mirror the evolutionary and functional composition of their underlying gene sets, or instead represent independent historical biases, remains unclear. Disentangling these dimensions requires integrating gene-level evolutionary properties with disease architecture and publication patterns.

Here, we systematically quantify research visibility across the human genome by stratifying genes according to PubMed publication counts and integrating standardized MANE Select annotations with evolutionary rate estimates, gene age assignments, sequence features, expression profiles, and disease ontologies. By mapping these features across visibility strata and disease categories, we characterize the multidimensional landscape underlying human gene prioritization. This framework allows us to ask whether highly studied genes occupy a constrained evolutionary and functional regime, whether understudied loci represent distinct biological territory, and how disease-level attention intersects with these intrinsic properties. Together, this analysis provides a quantitative basis for bias-aware and feasibility-aware research prioritization across genes and disease domains.

## Results

### PubMed publication counts per gene are heavy-tailed

**Fig. 1:**
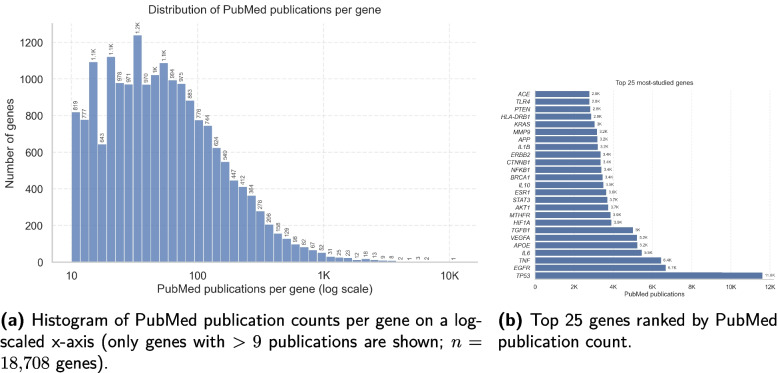
Genome-wide distribution of research attention. **a** PubMed publication counts per gene show a strongly right-skewed, heavy-tailed distribution spanning several orders of magnitude, indicating that most genes accumulate relatively few publications whereas a small subset dominates the literature. **b** The most highly studied genes correspond primarily to well-established signaling and disease-associated loci with exceptionally large publication counts

PubMed publication counts per gene exhibit a strongly right-skewed distribution spanning several orders of magnitude (Fig. 1A). While most genes accumulate only tens to low hundreds of publications, a small subset of genes is associated with thousands to more than ten thousand publications. The most-studied genes correspond to well-established disease and signaling hubs (Fig. 1B), illustrating a pronounced concentration of research effort.

### Highly studied genes cluster in GC content and low-visibility genes show increased density at shorter CDS lengths

To assess whether research attention is associated with intrinsic sequence features, we compared coding sequence (CDS) length and mRNA GC content across four gene sets: highly studied genes (*top500*), genes near rank 10,000 by publication count (*lowest500*), and two random control sets drawn from above and below rank 10,000. We defined rank 10,000 as a pragmatic midpoint separating highly characterized from sparsely characterized genes (see Methods). All sequence features were computed using MANE Select transcripts to ensure consistent transcript and protein definitions.

**Fig. 2:**
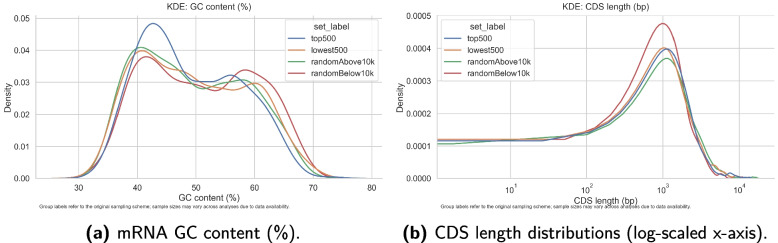
Sequence-feature distributions across research-attention strata (MANE Select). Kernel density estimates are shown for four gene groups defined at the sampling stage (top500, lowest500, randomAbove10k, randomBelow10k). **a** mRNA GC content distributions show that highly studied genes (*top500*) exhibit a somewhat sharper peak, indicating modest concentration, but largely overlap with the other groups. **b** CDS length distributions (log-scaled x-axis) show that low-visibility genes exhibit increased density at shorter CDS lengths, although the overall distributions overlap substantially across groups. Across both panels, differences arise primarily from changes in distribution shape and tail behavior rather than uniform shifts in central tendency. Sample sizes vary across analyses due to data availability (CDS length: top500 $n=491$, lowest500 $n=472$, randomAbove10k $n=475$, randomBelow10k $n=451$; GC content: top500 $n=493$, lowest500 $n=472$, randomAbove10k $n=475$, randomBelow10k $n=452$). Abbreviations: KDE, kernel density estimate; GC content, guanine–cytosine content; CDS length, coding sequence length; bw_adjust, bandwidth scaling parameter controlling the smoothness of KDE curves

Kernel density estimates reveal systematic differences in distribution shape. Highly studied genes (*top500*) show a somewhat sharper peak in GC content around 42–44%, indicating a modest concentration within a narrower range, but the majority of genes overlap substantially with the other groups (Fig. 2A). In contrast, low-visibility genes exhibit a broader distribution. For CDS length, low-visibility genes show increased density around shorter coding sequence lengths, whereas the overall distributions otherwise overlap substantially across groups (Fig. 2B). Overall, these differences primarily reflect changes in distribution shape and tail behavior rather than strong shifts in typical values.

### Evolutionary constraint differs across research-attention strata

 *Evolutionary rates in protein coding genes*. To test whether genes that differ in research attention also differ in evolutionary constraint, we analyzed pairwise coding sequence divergence between human and rhesus macaque. For each gene, nonsynonymous substitution rates (dN), synonymous substitution rates (dS), and their ratio (dN/dS) were estimated using pairwise maximum likelihood analyses in codeml. Analyses were restricted to genes for which both species sequences were available in the Kosiol et al. alignments [13], resulting in varying gene numbers across groups. This primarily reflects differences in orthology assignment and alignment quality rather than inconsistencies in gene selection. In particular, genes lacking identifiable orthologs or reliable sequence alignments—more common in the outgroup due to lower genome assembly quality at the time—could not be included in dN/dS estimation.

**Fig. 3:**
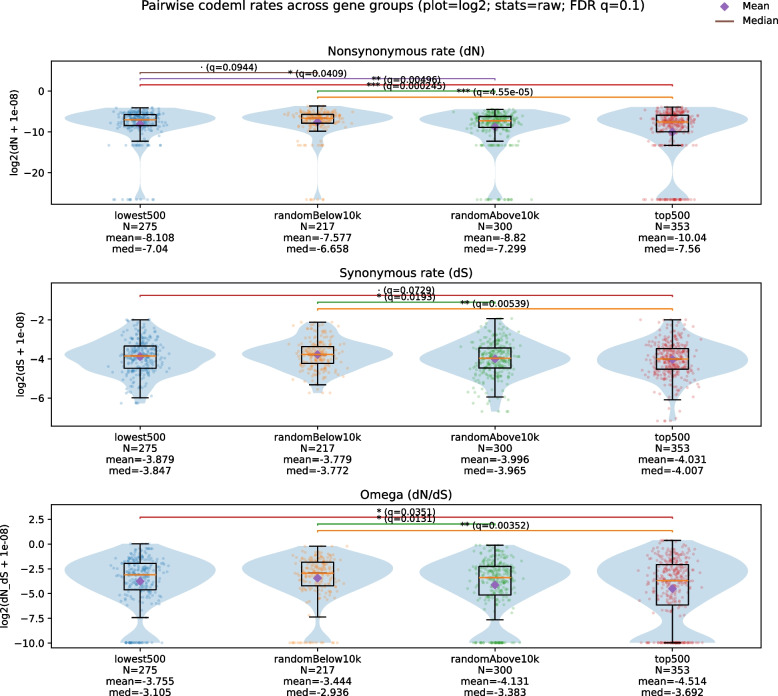
Evolutionary divergence across research-attention strata. Pairwise dN, dS, and dN/dS estimates between human (hg18) and rhesus macaque were obtained using codeml in pairwise mode based on alignments from Kosiol et al. [13]. Values are shown on a log$_2$ scale for visualization, whereas statistical tests were performed on the original (untransformed) rate estimates. Gene numbers vary across groups because not all genes could be assigned alignments in both species. All groups exhibit dN/dS values well below 1, consistent with purifying selection. However, genes sampled from below rank 10,000 show elevated dN and dN/dS relative to highly studied genes, indicating moderately reduced evolutionary constraint. Abbreviations: dN, nonsynonymous substitution rate (amino acid–changing substitutions per site); dS, synonymous substitution rate (silent substitutions per site); dN/dS, ratio of nonsynonymous to synonymous substitution rates, used as a proxy for selective constraint; randomBelow10k, genes randomly sampled from ranks below 10,000 with at least 10 publications

Across all four gene sets, median dN/dS values were well below 1, consistent with pervasive purifying selection and varying levels of genetic drift. However, distributions differed significantly among groups (Fig. 3).

Highly studied genes (*top500*) showed the lowest median dN/dS (0.0774; log$_2$ = $-3.69$), whereas genes drawn randomly from below rank 10,000 (*randomBelow10k*) showed higher ratios (median 0.1307; log$_2$ = $-2.94$). Genes near rank 10,000 and random genes above rank 10,000 showed intermediate values (medians 0.1162 and 0.0959; log$_2$ = $-3.11$ and $-3.38$, respectively). This corresponds to an approximately 1.7-fold higher median dN/dS in randomBelow10k genes compared to top500 genes on the raw scale. Nonparametric pairwise comparisons revealed significantly elevated dN/dS in randomBelow10k relative to top500 genes (FDR q = 3.52 $\times$ 10$^{-3}$) and relative to randomAbove10k genes (q = 0.0131).

Patterns in dN largely mirrored those observed for dN/dS. RandomBelow10k genes exhibited significantly higher dN than top500 genes (median log$_2$(dN + 1e-08): $-6.66$ vs. $-7.56$; q = 4.55 $\times$ 10$^{-5}$) and randomAbove10k genes ($-6.66$ vs. $-7.30$; q = 2.45 $\times$ 10$^{-4}$). In contrast, dS differences were more modest, though randomBelow10k genes showed significantly elevated dS compared to top500 genes (median log$_2$(dS + 1e-08): $-3.77$ vs. $-4.01$; q = 5.39 $\times$ 10$^{-3}$).

For visualization, rate estimates in Fig. 3 are shown on a log$_2$ scale, whereas all statistical comparisons and fold-change interpretations were performed using the original untransformed values. Together, these results indicate that genes receiving the greatest research attention tend to be under stronger long-term evolutionary constraint, whereas less visible genes, particularly those sampled from below rank 10,000, show moderately relaxed constraint. Importantly, all groups remain within a regime of strong purifying selection, suggesting quantitative rather than qualitative differences in evolutionary pressure.

*Positive selection.* To test whether research attention is associated with episodic adaptive evolution, we examined genes identified as FDR-significant in the “all branches” positive selection set from Kosiol et al [13]. Across all groups, only a small fraction of genes showed evidence of positive selection: 1.5% in lowest500 (5/328), 0.8% in randomAbove10k (3/355), 2.0% in randomBelow10k (5/256), and 2.7% in top500 (11/409). A chi-square test revealed no significant association between research-attention stratum and positive selection status ($\chi ^2 = 3.87$, $df = 3$, $p = 0.28$). Thus, unlike the systematic differences observed for dN and dN/dS, signatures of episodic positive selection are rare and do not vary detectably across research-attention strata, indicating that research bias is more closely associated with long-term purifying constraint than with adaptive evolution.

### Low-visibility genes are enriched for young gene ages

**Fig. 4:**
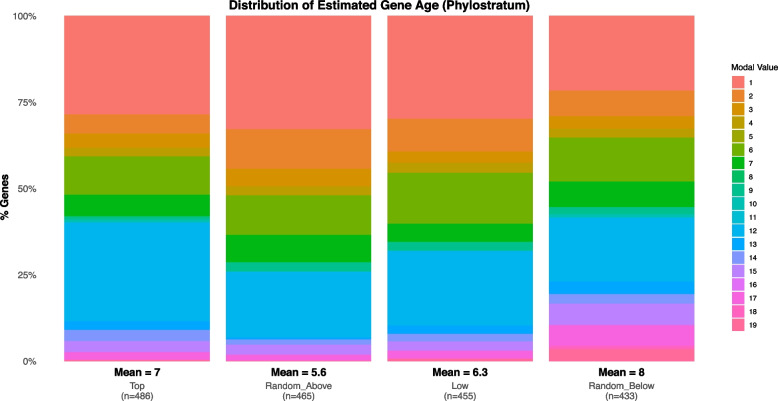
Distribution of estimated gene ages across research-attention strata. Gene ages were assigned using phylostratum annotations from Litman and Stein (19 phylostrata). Stacked bars show the percentage of genes per group assigned to each modal phylostratum category, where lower phylostratum values correspond to older evolutionary origins and higher values correspond to younger gene ages. Group means (phylostratum index) are shown above each bar. The randomBelow10k group is enriched for young genes and has the highest mean phylostratum (mean = 8, $n = 433$), whereas the randomAbove10k group shows the oldest profile (mean = 5.6, $n = 465$). The top500 and lowest500 groups show intermediate profiles (top500: mean = 7, $n = 486$; lowest500: mean = 6.3, $n = 455$)

To test whether research attention is associated with gene age, we annotated genes with phylostratum age estimates from Litman and Stein, which assigns human genes to 19 phylostrata [14]. We then compared the phylostratum composition across the four research-attention groups using stacked bar charts (Fig. 4).

The randomBelow10k group contains proportionally more genes assigned to young phylostrata and has the highest average gene age estimate (mean phylostratum = 8, $n = 433$). In contrast, the randomAbove10k group shows the fewest young genes and the oldest overall profile, with the lowest mean phylostratum (mean = 5.6, $n = 465$). The top500 group shows an intermediate distribution (mean = 7, $n = 486$), while the lowest500 group is also intermediate but slightly younger than top500 (mean = 6.3, $n = 455$). Together, these results indicate that reduced research attention is associated with a shift toward evolutionarily younger genes, whereas genes sampled from above rank 10,000 are enriched for older phylostrata.

Given that gene age and evolutionary constraint are often correlated, we next tested whether the observed differences in phylostratum composition were statistically robust across research-attention groups.

To formally assess differences in gene age distributions across research-attention strata, we performed a Kruskal–Wallis test, which revealed a significant overall effect of gene set on phylostratum assignment ($\chi ^2 = 43.75$, $df = 3$, $p < 0.001$). Pairwise Wilcoxon rank-sum tests with Benjamini–Hochberg correction showed that highly studied genes differed significantly from both the randomAbove10k set ($p = 0.0003$) and the randomBelow10k set ($p = 0.0069$), but not from the lowest500 gene set ($p = 0.063$). The randomBelow10k group displayed the most distinct distribution, differing significantly from highly studied genes ($p = 0.0069$), randomAbove10k genes ($p < 0.001$), and lowest500 genes ($p = 2.3 \times 10^{-5}$). In contrast, randomAbove10k and lowest500 genes were not significantly different ($p = 0.063$).

Together, these statistical comparisons confirm that gene age distributions vary systematically across research-attention strata. In particular, genes sampled from below rank 10,000 exhibit broader age distributions and are enriched for evolutionarily younger phylostrata, whereas highly studied genes tend to occupy older evolutionary categories, with substantial overlap between randomAbove10k and lowest500 groups.

### Low-visibility genes show reduced expression magnitude and increased tissue specificity

To determine whether research attention is associated with gene expression characteristics (https://www.proteinatlas.org/humanproteome/tissue/data#consensus_tissues_rna), we compared mean expression, maximum expression, and tissue specificity ($\tau$) across the four research-attention strata. Expression values were derived from consensus RNA-seq measurements (nTPM), and analyses were restricted to genes with a maximum expression of at least 1 nTPM in at least one tissue to exclude extremely low-expression loci [15].

**Fig. 5:**
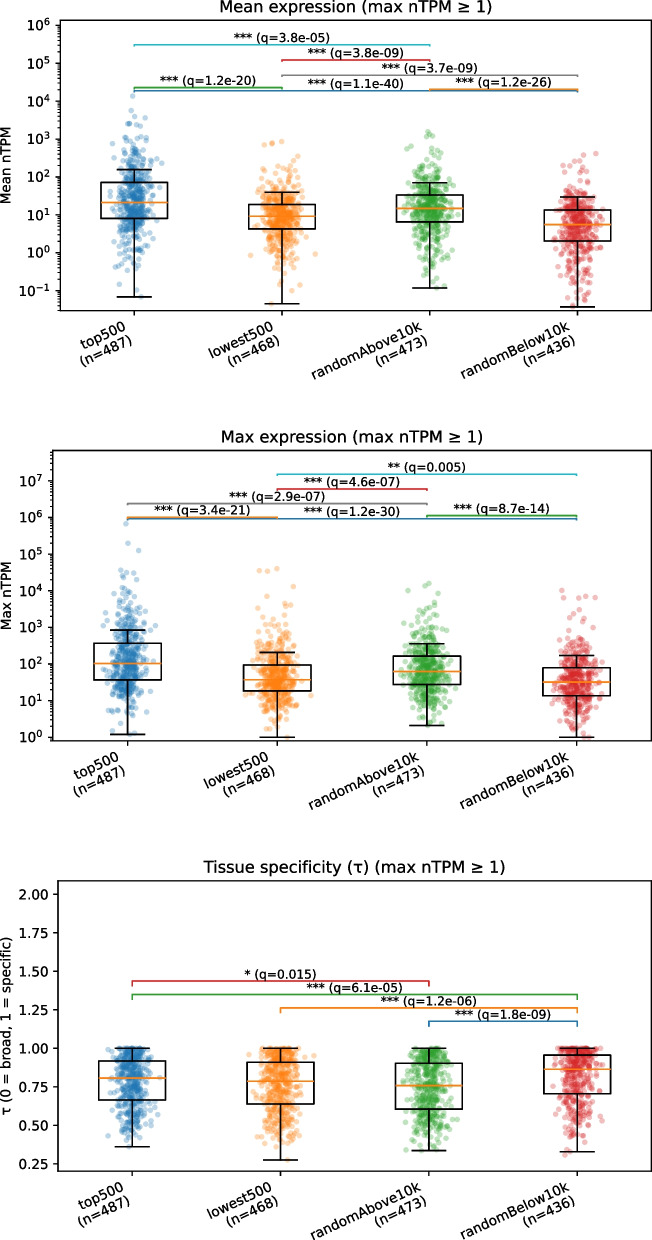
Gene expression characteristics across research-attention strata. Mean expression (left) and maximum expression (center) are shown on a log scale, and tissue specificity ($\tau$) is shown at right. Analyses were restricted to genes with detectable expression (at least 1 nTPM in at least one tissue). The number of genes included per group after filtering was: top500 ($n=487$), lowest500 ($n=468$), randomAbove10k ($n=473$), and randomBelow10k ($n=436$) (from initial sets of 491, 470, 475, and 450 genes, respectively). Boxes represent interquartile ranges, center lines medians, and whiskers 1.5$\times$IQR. Pairwise comparisons were performed using two-sided Mann–Whitney U tests with Benjamini–Hochberg correction

Expression magnitude differed markedly across groups (Fig. 5). Genes sampled from below rank 10,000 (randomBelow10k) exhibited the lowest mean expression (median = 5.51 nTPM, $n=436$), substantially below lowest500 (9.17), randomAbove10k (14.85), and top500 genes (21.1). All pairwise comparisons for mean expression were significant after FDR correction (all $q \le 3.81 \times 10^{-5}$), with the strongest contrast observed between randomBelow10k and top500 genes ($q = 1.14 \times 10^{-40}$).

Maximum expression followed a similar pattern. RandomBelow10k genes showed the lowest median maximum expression (32.15 nTPM), compared to lowest500 (37.2), randomAbove10k (62.8), and top500 genes (103.7). All pairwise comparisons were statistically significant following FDR correction ($q \le 5.13 \times 10^{-3}$), again with the largest separation between randomBelow10k and top500 genes ($q = 1.17 \times 10^{-30}$).

In contrast, tissue specificity showed the opposite trend. RandomBelow10k genes displayed the highest median $\tau$ (0.864), indicating greater tissue restriction, whereas randomAbove10k genes exhibited the lowest median $\tau$ (0.757). Top500 (median = 0.807) and lowest500 genes (median = 0.786) showed intermediate specificity. Pairwise comparisons revealed significant differences between randomBelow10k and all other groups (all $q \le 6.11 \times 10^{-5}$), whereas top500 and lowest500 genes did not differ significantly ($q = 0.243$).

Together, these results indicate that genes with the lowest research visibility are characterized by reduced overall expression and increased tissue specificity. Highly studied genes, by contrast, tend to exhibit broader and higher expression across tissues. These findings suggest that expression magnitude and breadth may represent intrinsic biological correlates of research attention.

### Correlated biological variables retain partially independent associations with research visibility

Because gene age, expression breadth, and evolutionary constraint are known to covary, we next assessed whether the observed relationships with research visibility remained detectable in a continuous multivariate framework. Results from the complementary multivariate analysis are summarized in Supplementary Table S1. Using genes with complete phylostratum and expression information ($n = 17{,}353$), we modeled log-transformed PubMed publication counts as a function of phylostratum age, mean expression, and tissue specificity ($\tau$).

Consistent with the stratified analyses, higher expression levels were associated with increased research visibility, whereas younger gene age and increased tissue specificity were associated with lower visibility. In the multivariate model, mean expression showed the strongest association with publication counts ($\beta = 0.209$, $p < 0.001$), whereas phylostratum retained a weaker but significant negative association ($\beta = -0.082$, $p < 0.001$). The effect of tissue specificity was substantially attenuated after accounting for the other variables, although it remained statistically significant ($\beta = -0.015$, $p < 0.001$).

Together, these results indicate that the evolutionary and expression-related features associated with low research visibility are partially correlated but not fully redundant.

### Research attention clusters around specific disease domains

To determine whether research bias extends beyond individual genes to disease contexts, we performed disease association enrichment analysis using STRING. This analysis was restricted to the top500 gene set, as no significant disease enrichments were detected for the other gene groups.

**Fig. 6:**
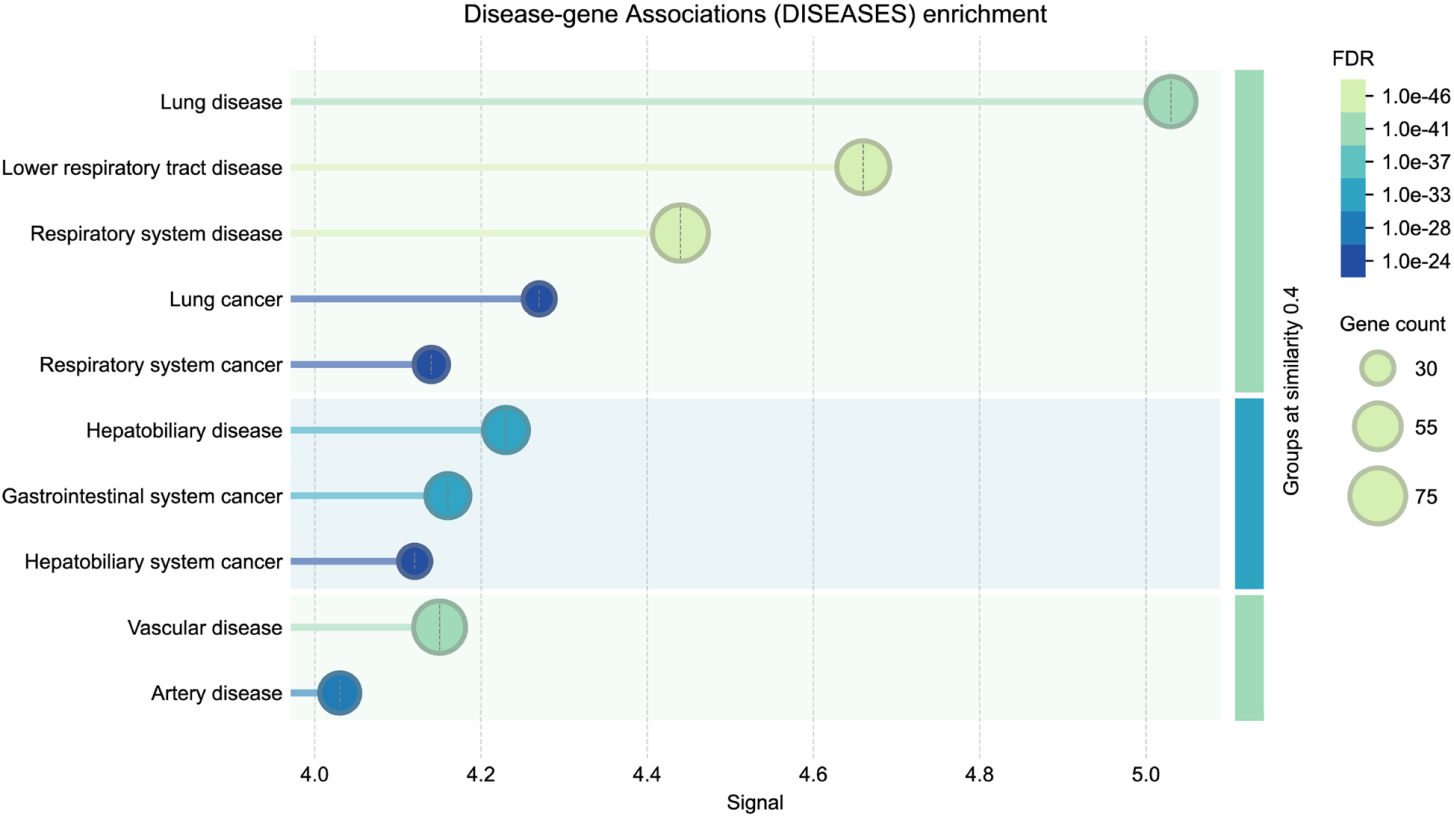
Disease association enrichment in highly studied genes. Network representation of enriched disease terms derived from STRING for the top500 gene set. Nodes represent disease terms and edges indicate semantic similarity greater than 0.4. Three major clusters correspond to respiratory diseases and lung cancer, gastrointestinal and hepatobiliary cancers, and vascular disease. No significant disease enrichment was observed for the other gene sets

Disease terms were clustered based on semantic similarity (threshold $>0.4$), yielding three coherent disease groups comprising ten enriched terms (Fig. 6).

The largest cluster was centered on respiratory diseases and lung cancer, including lung disease, lower respiratory tract disease, and respiratory system cancers. A second cluster comprised gastrointestinal and hepatobiliary disorders, including hepatobiliary disease and gastrointestinal system cancers. A third cluster represented vascular and arterial diseases.

These results indicate that highly studied genes are preferentially associated with a limited number of high-impact disease domains. This pattern was not observed for less-studied or randomly sampled gene sets, suggesting that disease-centric prioritization is a key driver of research attention.

### Disease-level research attention is highly uneven

We next asked whether these intrinsic evolutionary and functional properties propagate upward to the level of disease domains. To quantify research attention at the level of disease categories, we aggregated gene-level PubMed publication counts across disease–gene associations obtained from the DISEASES database. For each disease, research attention was summarized as the median number of PubMed publications across its associated genes. Analyses were restricted to diseases with at least ten genes that could be mapped to publication counts to ensure stable estimates.

**Fig. 7:**
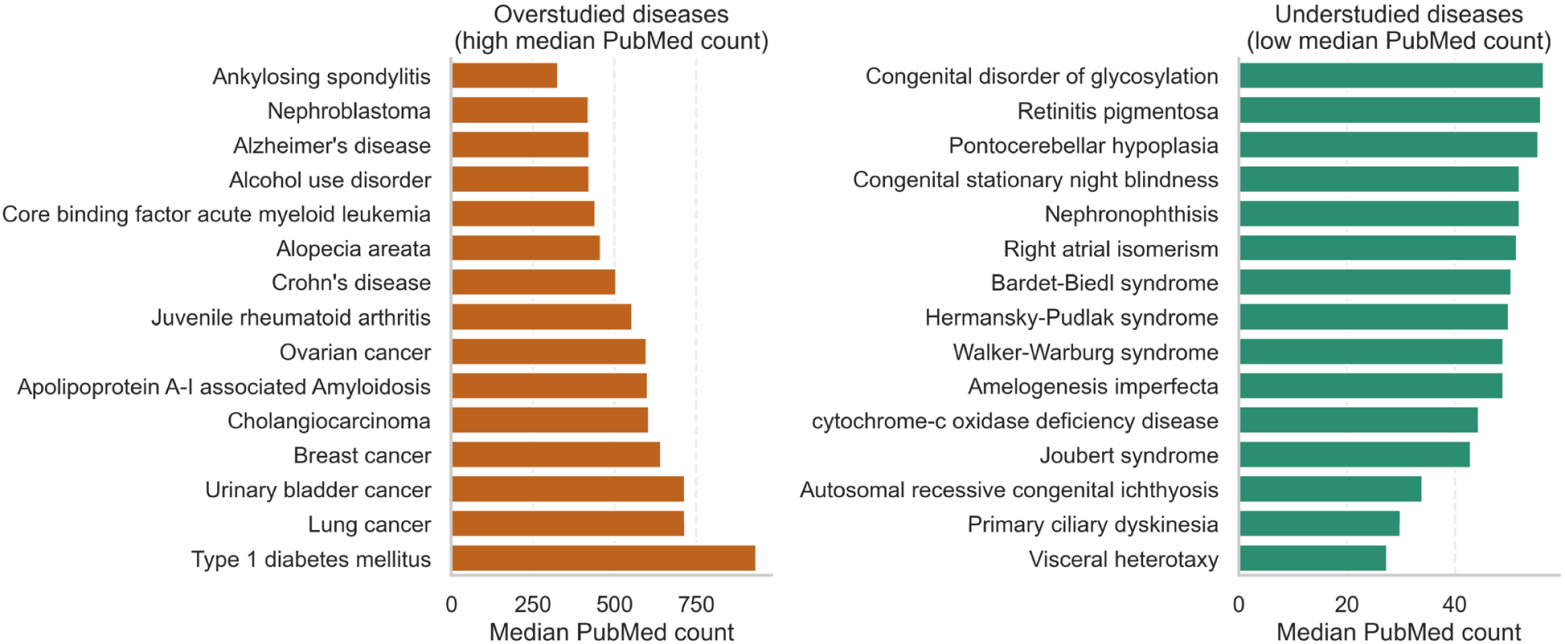
Disease-level research attention is highly uneven. Diseases were ranked by the median PubMed publication count of their associated genes. Overstudied diseases (left) are dominated by highly cited genes and largely correspond to cancer, inflammatory, and cardiovascular disease categories. Understudied diseases (right) are enriched for congenital, developmental, metabolic, mitochondrial, neuromuscular, and inherited retinal disorders and show uniformly low gene-level publication counts. Only diseases with at least ten genes mapped to PubMed publication counts were included

This analysis revealed a pronounced asymmetry in disease-level research attention (Fig. 7). Overstudied diseases were dominated by cancer, inflammatory, and cardiovascular disease categories and were characterized by high median publication counts across their associated genes. In contrast, the most understudied diseases were overwhelmingly enriched for congenital, developmental, metabolic, mitochondrial, neuromuscular, and inherited retinal disorders. These diseases exhibited uniformly low publication counts across their gene sets and frequently lacked any genes among the global top-500 most studied genes.

Together, these results demonstrate that research bias operates strongly at the level of disease domains, with gene-level research attention emerging, in part, as a downstream consequence of disease-centric prioritization.

### Orphan diseases are systematically underrepresented in gene-level research attention

To test whether disease rarity is associated with research attention, diseases were classified as orphan or non-orphan using the Orphanet rare disease classification. Analyses were restricted to diseases with at least ten associated genes that could be mapped to PubMed publication counts to ensure stable disease-level estimates. For each disease, research attention was summarized as the median PubMed publication count across its associated genes.

**Fig. 8:**
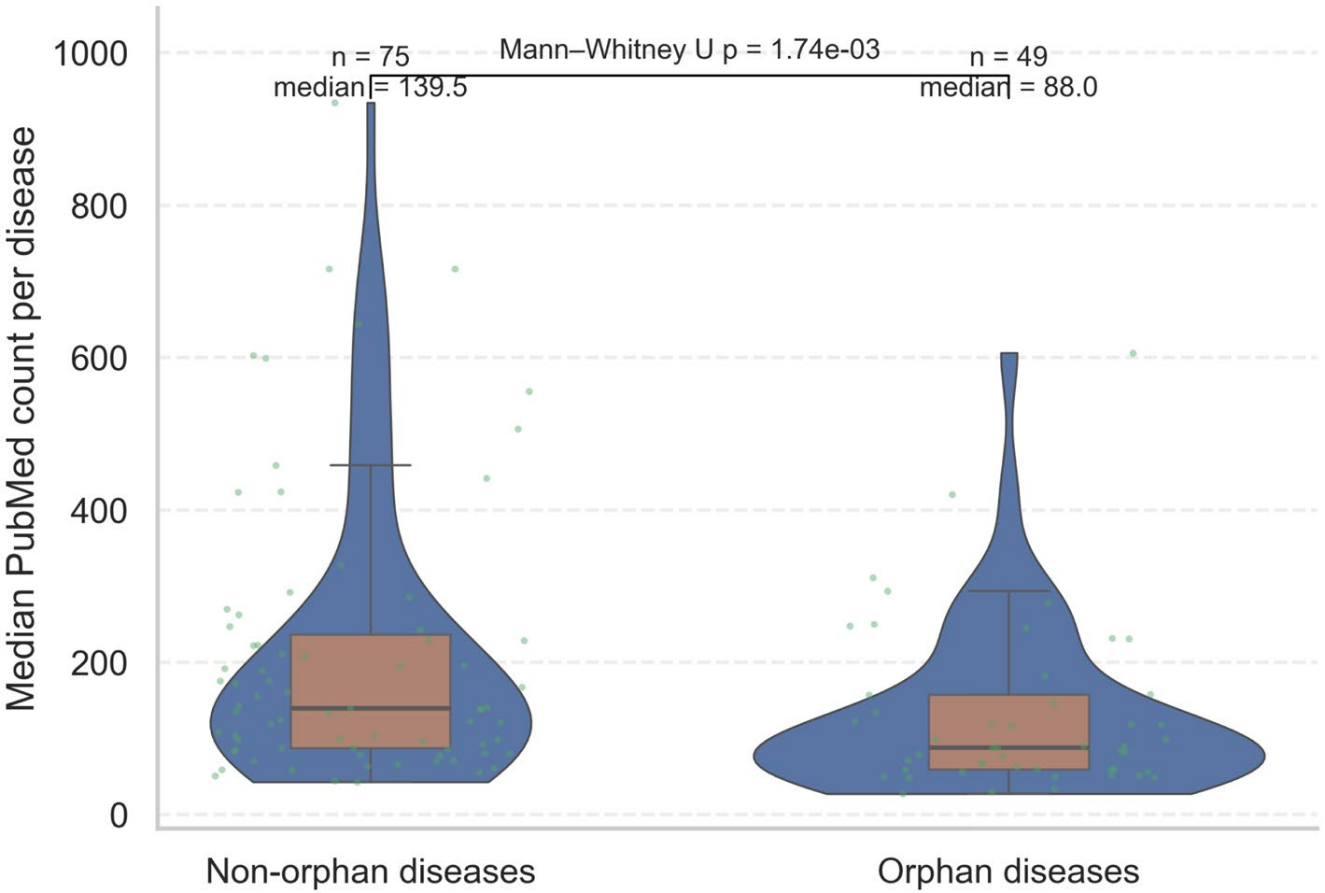
Orphan diseases are associated with lower gene-level research attention. Diseases were classified as orphan or non-orphan based on their presence in the Orphanet rare disease classification (December 2025 release). For each disease, research attention was summarized as the median PubMed publication count across associated genes (DISEASES knowledge channel, filtered), restricting to diseases with at least ten genes mapped to PubMed publication counts. Orphan diseases show significantly lower median publication counts than non-orphan diseases (Mann–Whitney U test, $p = 1.74 \times 10^{-3}$)

Orphan diseases showed significantly lower gene-level research attention than non-orphan diseases (Fig. 8). The median PubMed publication count per disease was 88.0 for orphan diseases compared to 139.5 for non-orphan diseases (Mann–Whitney U test, $p = 1.74 \times 10^{-3}$). These results indicate that disease rarity represents an additional axis along which research attention is structured, consistent with systematic underrepresentation of rare disease gene sets in the biomedical literature.

A concise synthesis of the principal contrasts between highly studied genes (top500) and low-visibility genes (randomBelow10k) across evolutionary, molecular, and functional dimensions is provided in Supplementary Table S2.

## Discussion

Biomedical research is often assumed to converge on the most biologically important genes and diseases. However, our results show that research attention across the human genome is highly structured and uneven, with a small subset of genes and disease domains receiving disproportionate focus while large portions remain understudied [16]. This bias does not simply reflect a continuum of biological relevance but instead reveals a distinct low-visibility gene stratum with characteristic properties.

Across multiple dimensions, understudied genes differ systematically from highly studied genes. They tend to exhibit greater heterogeneity in sequence composition, weaker evolutionary constraint, younger evolutionary origin, and lower and more tissue-specific expression. Together, these features are consistent with a class of genes that is more difficult to investigate using standard experimental and comparative approaches, including reliance on conserved orthologues, broad expression, and canonical model systems. In parallel, research attention at the disease level is concentrated within a limited number of major domains, while rarer and more specialized disease classes remain comparatively underexplored. These observations suggest that research bias is shaped not only by sociological factors but also by intrinsic properties that affect experimental tractability.

Because gene age, evolutionary constraint, and expression breadth are themselves correlated, the observed associations with research visibility should not be interpreted as fully independent biological signals. Complementary multivariate analyses nevertheless showed that gene age, expression magnitude, and tissue specificity retained partially independent associations with publication counts after accounting for covariation among predictors (Supplementary Table S1). In particular, expression magnitude showed the strongest relationship with research visibility, whereas the effect of tissue specificity was substantially attenuated in the joint model. These results suggest that the understudied genome is characterized by overlapping evolutionary and functional properties rather than by a single dominant feature.

These findings have important implications for gene discovery and prioritization. Commonly used features such as conservation, network connectivity, and expression breadth are themselves correlated with historical research attention, raising the possibility that current approaches may systematically overlook low-visibility genes. Addressing this bias will likely require both methodological adjustments and experimental strategies tailored to genes with limited conservation, narrow expression, or lineage-specific functions.

Several limitations should be acknowledged. Publication counts provide only an approximate proxy for research attention and do not distinguish between different forms of literature contribution, including primary discovery studies, reviews, methodological papers, clinical reports, or recurrent use of highly visible genes as reference markers. Consequently, highly cited genes such as *TP53* may accumulate publications partly through widespread reuse across diverse biomedical contexts rather than exclusively through direct functional characterization. Nevertheless, the highest-ranked genes recovered in our analysis correspond predominantly to canonical signaling, cancer, and disease-associated loci (e.g. *TP53*, *EGFR*, *TNF*, *VEGFA*), which are also consistently identified in previous meta-research studies of biomedical publication bias and gene-level research concentration [1, 4]. Although publication counts do not directly measure depth of mechanistic understanding, they provide a useful large-scale proxy for sustained biomedical research attention and community focus.

In addition, disease-gene association analyses remain susceptible to indirect forms of circularity. Although text-mining-derived associations were excluded from the DISEASES database analysis to minimize direct coupling between publication counts and disease annotations, curated disease resources are themselves influenced by historical study intensity. Genes receiving sustained experimental attention are therefore more likely to accumulate disease annotations over time, potentially reinforcing existing visibility biases within disease-level analyses. Accordingly, the disease-enrichment results presented here should be interpreted primarily as describing how research attention is structured across disease domains rather than as independent validation of biological importance.

Additional limitations arise from the evolutionary analyses, which depend on the availability of high-confidence orthologues and alignments. This may preferentially exclude younger or rapidly evolving genes, particularly in more weakly assembled outgroup genomes. Finally, the stratified analyses used here were intended primarily as an interpretable framework for contrasting highly studied and low-visibility regions of the publication landscape rather than as sharp biological categories. Indeed, publication-count resolution becomes increasingly compressed among low-ranked genes, where many loci share identical or near-identical publication counts (Supplementary Table S3).

Despite these caveats, the consistent patterns observed across sequence, evolutionary, expression, and disease-level analyses indicate that low-visibility genes occupy a biologically distinct and still underexplored region of the genome. Systematic investigation of this space is therefore likely to uncover mechanisms and disease relationships that remain poorly captured by current research priorities.

## Conclusion

Using complementary sequence, evolutionary, expression, and disease-level analyses, we show that biomedical research attention across the human genome is highly structured and uneven. Highly studied genes tend to be evolutionarily older, more constrained, and more broadly expressed, whereas low-visibility genes are enriched for younger, more tissue-specific, and moderately less constrained loci. These findings indicate that the understudied genome represents a biologically distinct and systematically underexplored region of human gene space.

Our results further suggest that current research priorities are shaped not only by sociological reinforcement but also by intrinsic biological properties that influence experimental tractability. Because annotation density, conservation, and network connectivity are themselves entangled with research history, bias-aware prioritization strategies will be important for expanding systematic discovery, particularly for understudied genes and rare disease biology [17].

## Methods

### Gene ranking and set construction

Human genes were ranked by PubMed publication count using NCBI-derived data restricted to *Homo sapiens*.

Four gene sets of equal size ($n = 500$ per group prior to downstream filtering) were constructed using a combination of rank-based selection and random sampling. Specifically, the *top500* group comprises the 500 highest-ranked genes by publication count, representing the most intensively studied loci. The *lowest500* group comprises genes centered around rank 10,000, providing a reference point near the midpoint of the heavy-tailed distribution.

To provide appropriate controls, we additionally constructed two randomly sampled groups: *randomAbove10k*, consisting of genes drawn uniformly at random from ranks 1–10,000 (excluding the top500 set), and *randomBelow10k*, consisting of genes drawn randomly from ranks below 10,000 with at least 10 publications. These random sets capture the broader distributions above and below the threshold, allowing us to distinguish local rank effects from global differences between well-studied and low-visibility genes.

The threshold at rank 10,000 approximates the midpoint of the heavy-tailed distribution and provides a pragmatic separation between more intensively studied and sparsely characterized genes (Fig. 1). Rank 10,000 was selected as a pragmatic compromise for separating more highly studied genes from lower-visibility genes. To assess how informative exact rank position remained across the publication-count distribution, we evaluated fixed 500-gene windows at different rank intervals. This analysis showed that lower-ranked regions contained progressively fewer distinct PubMed-count values, larger tie blocks, and increasing numbers of genes outside the focal window with publication counts identical to genes inside the window (Supplementary Table S3). Thus, rank 10,000 was used as a practical threshold rather than as a sharp biological or bibliometric boundary.

For consistency, we use shorthand labels (top500, lowest500, randomAbove10k, randomBelow10k) to refer to the four gene groups defined at the sampling stage. Due to data availability in downstream analyses (e.g., missing alignments or expression values), the number of genes included per group varies across figures. Exact sample sizes are therefore reported in each figure legend.

Throughout the manuscript, the terms “highly studied genes” and “top500 genes” are used interchangeably to refer to the same gene set comprising the 500 genes with the highest PubMed publication counts. Likewise, “low-visibility genes” refers primarily to the randomBelow10k group unless otherwise stated explicitly.

### MANE Select transcript annotation

MANE Select annotations were used to define a single representative transcript and protein per gene [18]. Corresponding RefSeq mRNA and protein sequences were used for all sequence feature calculations.

### Sequence feature calculation

CDS length was inferred as three times the MANE Select protein length. mRNA GC content was calculated as the fraction of guanine and cytosine nucleotides among all standard bases. Kernel density estimates were computed using a shared bandwidth adjustment to facilitate comparison across gene sets.

### Pairwise evolutionary rate estimation

To assess evolutionary constraint, coding sequence divergence between human and rhesus macaque [19] was estimated using codeml from the PAML package [20] in pairwise mode. Protein-coding alignments were obtained from the dataset of Kosiol et al. [13], restricting analyses to orthologous human (hg18) and rhesus macaque (rheMac2) sequences when available. We used these data without remapping to newer genome builds in order to maintain full consistency with the original alignment framework and the associated evolutionary rate estimates. While more recent genome assemblies (e.g., GRCh38) are available, re-deriving genome-wide, codon-aware multi-species alignments is non-trivial and may introduce methodological differences that confound direct comparison with the published results. Our approach therefore prioritizes comparability and reproducibility of previously established evolutionary inferences.

For each gene, dN, dS, and dN/dS were estimated under the standard codon substitution model implemented in codeml pairwise mode. Only pairwise comparisons were performed. dN/dS estimates were only available for genes with identifiable one-to-one orthologs across the species considered. Genes lacking confident orthology assignments or high-quality alignments—particularly in the outgroup genome—were excluded from this analysis.

Statistical comparisons across groups were conducted using nonparametric Mann–Whitney U tests for pairwise contrasts. Effect sizes were summarized using the Hodges–Lehmann estimator and Cliff’s delta. Multiple testing correction across pairwise comparisons was performed using the Benjamini–Hochberg false discovery rate procedure. All statistical analyses were conducted on raw rate estimates, while figures were visualized on a log$_2$ scale for comparability.

### Gene age annotation and phylostratum analysis

Gene age estimates were obtained from Litman and Stein [14], which assigns human genes to 19 discrete phylostrata based on evolutionary origin. For each gene in the four research-attention groups, we extracted the modal phylostratum (gene age category) value as the primary age category.

The modal phylostratum (gene age category) corresponds to the most likely inferred evolutionary origin assigned to each gene, with lower phylostratum indices representing older evolutionary origins and higher indices representing younger genes. Genes without an assigned phylostratum value were excluded from this analysis, resulting in varying group sample sizes.

To visualize group-level differences in gene age composition, we computed the proportion of genes per group in each phylostratum category and plotted these as stacked bar charts. In addition, we summarized each group using the mean phylostratum index as a compact measure of relative gene age, with higher values indicating younger inferred origins under this coding.

The distribution analysis of estimated age (phylostratum) was done in R (v. 4.4.1) using stacked bar charts. RStudio (v. 2025.9.2.418) was used as the development environment.

### Gene expression analysis

Gene-level RNA expression values were obtained from the consensus tissue expression dataset (rna_tissue_consensus.tsv), which reports normalized transcripts per million (nTPM) across human tissues [21, 22]. The Human Protein Atlas reports consensus-normalized expression values as normalized transcripts per million (nTPM), which are batch-corrected TPM estimates integrated across datasets.

For each gene, three summary metrics were computed:

(i) mean expression across all tissues,
(ii) maximum expression across tissues, and
(iii) tissue specificity ($\tau$).

The tissue specificity index was calculated following Yanai et al. [23]:

$$\begin{aligned} \tau = \frac{\sum _{i=1}^{n} \left( 1 - \frac{x_i}{\max (x)}\right) }{n-1}, \end{aligned}$$

where $x_i$ denotes expression in tissue *i*, and *n* is the number of tissues. $\tau$ ranges from 0 (uniform expression across tissues) to 1 (expression restricted to a single tissue).

To exclude extremely low-expression loci and avoid instability in specificity estimates, analyses were restricted to genes with a maximum expression of at least 1 nTPM in at least one tissue.

Genes were assigned to four predefined research-attention strata (top500, lowest500, randomAbove10k, randomBelow10k). Group comparisons were performed using two-sided Mann–Whitney U tests. *P*-values were adjusted for multiple testing using the Benjamini–Hochberg false discovery rate (FDR) procedure. An FDR-adjusted threshold of $q \le 0.05$ was considered statistically significant.

### Multivariate analysis of research visibility

To assess whether gene age and expression-related variables retained independent associations with research visibility, we performed complementary multivariate analyses across all genes with complete data availability. Gene-level PubMed publication counts were log$_{10}$-transformed after adding a pseudocount of 1. Predictors included phylostratum age, mean expression, and tissue specificity ($\tau$). Mean expression values were log$_{10}$-transformed prior to analysis. Tissue specificity was recalculated directly from tissue-level expression profiles following Yanai et al. [23]. Analyses were restricted to genes with detectable expression in at least one tissue (maximum expression $\ge 1$ nTPM). Predictors were standardized before fitting ordinary least-squares regression models to facilitate comparison of effect sizes.

### Visualisation

For visualization, mean and maximum expression values were plotted on a logarithmic scale. Boxplots display medians, interquartile ranges, and whiskers extending to 1.5$\times$IQR. All analyses were conducted in Python (SciPy and statsmodels) unless otherwise stated.

### Disease association enrichment

Disease association enrichment was performed using STRING with standard parameters [24]. The analysis was conducted on the top500 gene set using HGNC gene symbols as input. No significant disease enrichments were detected for the other gene groups and they were therefore not considered further.

Significantly enriched disease terms were clustered based on semantic similarity, and only relationships with similarity greater than 0.4 were retained for visualization and interpretation.

### Disease-level aggregation of research attention

Disease–gene associations were obtained from the DISEASES database (JensenLab) using the filtered Knowledge channel, which integrates curated databases and genome-wide association studies [25]. Text-mining-derived associations were excluded to avoid circularity with publication-based gene ranking.

Gene-level research attention was quantified using PubMed publication counts. When multiple records mapped to the same gene symbol, the maximum publication count was retained. For each disease, publication counts were aggregated across associated genes, and the median PubMed count was used as a robust summary statistic.

Analyses and visualizations were restricted to diseases with at least ten associated genes that could be mapped to publication counts, to avoid unstable estimates driven by very small gene sets. Diseases were ranked by their median gene-level publication count to identify overstudied and understudied disease categories.

### Disease rarity classification

Diseases were classified as orphan or non-orphan using the Orphanet rare disease classification obtained from Orphadata (December 2025 release), an ELIXIR Core Data Resource and Global Core Biodata Resource [26]. The Orphanet Scientific Knowledge Base provides a curated reference nomenclature for rare diseases and is widely used for rare disease research and clinical annotation. All domain-specific Orphanet classifications of rare diseases were combined to generate a comprehensive reference set. Diseases present in the Orphanet classification were labeled as orphan diseases, while all others were classified as non-orphan. Disease name matching was performed conservatively using normalized string matching, and ambiguous matches were excluded from downstream analyses.

## Supplementary Information

Supplementary Material 1.

## Data Availability

All analytical scripts, processed datasets, and derived summary tables are publicly available in the GitHub repository: https://github.com/eswaeswaran/genevisibility. This repository contains the code and processed data required to reproduce the analyses presented in this study.

## Abbreviations

CDS: Coding sequence
DISEASES: Disease–gene association database from JensenLab
dN: Nonsynonymous substitution rate
dS: Synonymous substitution rate
FDR: False discovery rate
GC: Guanine-cytosine
MANE: Matched Annotation from NCBI and EMBL-EBI
nTPM: normalized transcripts per million
STRING: Search Tool for the Retrieval of Interacting Genes/Proteins
