# Supplementary material for: Evolutionary and functional constraints structure human gene research visibility
Source: BMC Genomics. 2026 Jun 3;27:526. doi: 10.1186/s12864-026-13001-5 (PMC13244977; doi:10.1186/s12864-026-13001-5)
Supplement: Supplementary file 1 — Supplementary Material 1. [file 12864_2026_13001_MOESM1_ESM.pdf]

Supplementary Material

Table S1: Multivariate regression analysis of factors associated with research visibility. Ordinary least-squares regression was performed using $\log_{10}$(PubMed publication count + 1) as the response variable. Predictors were standardized prior to analysis to facilitate comparison of effect sizes. Analyses were restricted to genes with complete phylostratum and expression information and detectable expression in at least one tissue ($n = 17{,}353$).

Predictor	Coefficient (β)	Std. error	t value	p value
Intercept	1.7054	0.0032	525.66	$< 2 \times 10^{-16}$
Phylostratum	-0.0822	0.0035	-23.60	$< 2 \times 10^{-16}$
Mean expression ($\log_{10}$)	0.2089	0.0035	59.56	$< 2 \times 10^{-16}$
Tissue specificity (τ)	-0.0148	0.0036	-4.16	3.2×10^{-5}

Model statistics: $R^2 = 0.256$, adjusted $R^2 = 0.256$, F-statistic = 1987, $p < 2 \times 10^{-16}$. Higher phylostratum values correspond to younger inferred gene ages.

Table S2: Conceptual contrast between highly studied genes and low-visibility genes. This table summarizes the principal qualitative differences between the two most contrasting research-attention strata (top500 and randomBelow10k) across evolutionary, molecular, and functional dimensions, integrating observations from Figs. **??**, **??**, and **??**. Intermediate groups are not shown for clarity.

**Feature**	**Top500**	**RandomBelow10k**
Research attention	Highly studied; dominant in literature	Low visibility; below rank 10,000
Evolutionary constraint	Stronger long-term constraint	Moderately relaxed constraint; heavier upper tail
Gene age	Intermediate to older profile	Enriched for younger, lineage-restricted genes
Expression profile	Higher expression; broader across tissues	Lower expression; increased tissue specificity
Sequence features	Narrow GC-content regime; not depleted of long CDSs	Broader GC distribution; increased density of shorter CDSs
Experimental tractability	Strong cross-species homology; amenable to model systems	Reduced homology; context-dependent expression
Methodological implication	Well captured by standard comparative and network-based approaches	Likely underdetected by standard pipelines; requires context-specific and human-relevant systems
High-level interpretation	Conserved, constrained genes with extensive functional characterization	Distinct class of younger, less constrained genes that remain systematically underexplored

Table S3: Loss of rank resolution across lower-visibility gene regions. Fixed 500-gene windows were evaluated across different regions of the PubMed publication-count ranking. Lower-ranked windows show increasingly compressed publication-count distributions, with fewer distinct count values, larger tie blocks, and more genes outside the focal window sharing identical publication counts. "Equivalent genes outside window" denotes genes excluded from the focal 500-gene window despite having publication counts identical to genes within that window.

Window	Rank start	Rank end	Min count	Max count	Range	Unique counts	Max tie size	Equivalent genes outside window
top500	1	500	518	11,608	11,090	383	4	0
ranks_4501_5000	4,501	5,000	97	107	10	11	60	50
ranks_9501_10000	9,501	10,000	43	47	4	5	148	225
ranks_14501_15000	14,501	15,000	20	22	2	3	281	328
ranks_19501_20000	19,501	20,000	7	8	1	2	376	525

Table S4: Public data sources used in this study. For each resource, the table lists the dataset or file used, its role in the analysis, the source repository/database, and a direct web link.

Analysis component	Dataset / file	Repository / database	Identifier / version	Direct link
Gene model definition / representative transcript selection	MANE Select transcript set	Matched Annotation from NCBI and EMBL-EBI (MANE)	MANE GRCh38 v1.5 (MANE.GRCh38.v1.5.summary.txt)	https://www.ncbi.nlm.nih.gov/refseq/MANE/
Transcript and protein sequence retrieval	RefSeq mRNA and protein sequences corresponding to MANE Select transcripts	NCBI Reference Sequence Database (RefSeq)	See processed table	https://www.ncbi.nlm.nih.gov/refseq/
Gene-level publication ranking	Gene–PubMed publication count mapping (gene2pubmed.gz)	NCBI Gene / PubMed	Downloaded 2025; includes all Gene IDs linked to PubMed (no symbol-based aggregation)	https://ftp.ncbi.nlm.nih.gov/gene/DATA/
Expression analysis	rna_tissue_consensus.tsv	The Human Protein Atlas	HPA v24 (downloaded 18 Feb 2026)	https://www.proteinatlas.org/about/download
Disease–gene association analysis	human_disease_knowledge_filtered.tsv; human_disease_knowledge_full.tsv	DISEASES database (JensenLab)	Downloaded 2025 (files: filtered and full knowledge datasets)	https://diseases.jensenlab.org/Downloads
Disease resource landing page	Disease–gene association database	DISEASES database (JensenLab)	Not applicable	https://diseases.jensenlab.org/
Rare disease / orphan classification	Orphanet classification files	Orphadata / Orphanet	Dec 2025 release (downloaded 10 Dec 2025)	https://www.orphadata.com/_classifications/
Rare disease classification landing page	Rare disease classification browser	Orphanet	Not applicable	https://www.orpha.net/en/disease/classification

Continued on next page

Supplementary Table continued.

Analysis component	Dataset / file	Repository / database	Identifier / version	Direct link
Evolutionary rate analysis	Human–rhesus macaque alignments (Kosiol et al.)	Mammalian positive selection dataset (Kosiol et al.)	As reported in [13] (accessed 2025)	`http://compgen.bscb.cornell.edu/projects/mammal-psg/`
Gene age analysis	Phylostratum assignments	Published dataset (Litman and Stein)	As reported in [14] (Supplementary dataset)	See supplementary materials of cited publication
Code, processed data, and derived tables	Analysis scripts and processed datasets	Private repository (review access)	Review-access repository	`https://tu-dortmund.sciebo.de/s/GroQkLZNFWZTG8H`

References

[1] Elie Dolgin. The most popular genes in the human genome. *Nature*, 551(7681):427–431, November 2017.

[2] Ian Dunham. Human genes: Time to follow the roads less traveled? *PLOS Biology*, 16(9):e3000034, September 2018.

[3] Winston A. Haynes, Aurelie Tomczak, and Purvesh Khatri. Gene annotation bias impedes biomedical research. *Scientific Reports*, 8(1), January 2018.

[4] Thomas Stoeger, Martin Gerlach, Richard I. Morimoto, and Luís A. Nunes Amaral. Large-scale investigation of the reasons why potentially important genes are ignored. *PLOS Biology*, 16(9):e2006643, September 2018.

[5] Jennifer A Byrne, Yasunori Park, Reese A K Richardson, Pranujan Pathmendra, Mengyi Sun, and Thomas Stoeger. Protection of the human gene research literature from contract cheating organizations known as research paper mills. *Nucleic Acids Research*, 50(21):12058–12070, November 2022.

[6] Kuo-Feng Tung, Chao-Yu Pan, Chao-Hsin Chen, and Wen-chang Lin. Top-ranked expressed gene transcripts of human protein-coding genes investigated with gtex dataset. *Scientific Reports*, 10(1), October 2020.

[7] Reese Richardson, Heliodoro Tejedor Navarro, Luis A Nunes Amaral, and Thomas Stoeger. Meta-research: Understudied genes are lost in a leaky pipeline between genome-wide assays and reporting of results. *eLife*, 12, March 2024.

[8] Toni I. Gossmann, Peter D. Keightley, and Adam Eyre-Walker. The effect of variation in the effective population size on the rate of adaptive molecular evolution in eukaryotes. *Genome Biology and Evolution*, 4(5):658–667, 2012.

[9] Jianzhi Zhang and Jian-Rong Yang. Determinants of the rate of protein sequence evolution. *Nature Reviews Genetics*, 16(7):409–420, June 2015.

[10] Arina Afanasyeva, Mathias Bockwoldt, Christopher R. Cooney, Ines Heiland, and Toni I. Gossmann. Human long intrinsically disordered protein regions are frequent targets of positive selection. *Genome Research*, 28(7):975–982, June 2018.

[11] Jian-Hai Chen, Patrick Landback, Deanna Arsala, Alexander Guzzetta, Shengqian Xia, Jared Atlas, Dylan Sosa, Yong E. Zhang, Jingqiu Cheng, Bairong Shen, and Manyuan Long. Evolutionarily new genes in humans with disease phenotypes reveal functional enrichment patterns shaped by adaptive innovation and sexual selection. *Genome Research*, 35(3):379–392, February 2025.

[12] Erich Bornberg-Bauer and Lars A. Eicholt. Emergence and evolution of protein-coding de novo genes. *Nature Reviews Genetics*, January 2026.

[13] Carolin Kosiol, Tomáš Vinař, Rute R. da Fonseca, Melissa J. Hubisz, Carlos D. Bustamante, Rasmus Nielsen, and Adam Siepel. Patterns of positive selection in six mammalian genomes. *PLoS Genetics*, 4(8):e1000144, August 2008.

[14] Thomas Litman and Wilfred D. Stein. Obtaining estimates for the ages of all the protein-coding genes and most of the ontology-identified noncoding genes of the human genome, assigned to 19 phylostrata. *Seminars in Oncology*, 46(1):3–9, February 2019.

[15] María Bueno Álvez, Sofia Bergström, Josefin Kenrick, Emil Johansson, Mikael Åberg, Murat Akyildiz, Ozlem Altay, Hilda Sköld, Konstantinos Antonopoulos, Emmanouil Apostolakis, Yasin Hasan Balcioglu, Anna Bergström, Göran Bergström, Sophia Björkander, Suzanne Egyhazi Brage, Petter Brodin, Lynn Butler, Sara Cajander, Hanna Danielsson, Murat Dayangac, Gizem Dinler-Doganay, Levent Doğanay, Gunilla Enblad, Malin Enblad, Linn Fagerberg, Sara Falck-Jones, Anna Färnert, Mattias Forsberg, Laura Gonzalez, Anders Gummesson, Karin Gunnarsson, Iva Gunnarsson, Ulf Gyllensten, Göran Hesselager, Andreas Hober, Martin Höglund, Marie Holmqvist, Begum Horuluoglu, Rebecka Hultgren, Maria Jesus Iglesias, Helena Janols, Fredric Johansson, Anette Johnsson, Lars Klareskog, David Kotol, Inger Kull, Marika Kvarnström, Maximilian Julius Lautenbach, Ulrika Liljedahl, Henrik Lindman, Cecilia Lindskog, Miklos Lipcsey, Ingrid E. Lundberg, Adil Mardinoglu, Erik Melén, Lingqi Meng, Anne-Sophie Merritt, Jan Mulder, Mai Thi-Huyen Nguyen, Jessica Nordlund, Anna Norrby-Teglund, Antonella Notarnicola, Piotr Nowak, Jacob Odeberg, Per Oksvold, Tomas Olsson, Leonid Padyukov, Karlis Pauksens, Fredrik Piehl, Elisa Pin, Fredrik Pontén, Natallia Rameika, Anton Reepalu, Joy Roy, Jochen M. Schwenk, Meltem Sen, Antti Siika, Oscar E. Simonson, Åsa Sivertsson, Tobias Sjöblom, Evelina Sjöstedt, Lovisa Skoglund, Anna Smed-Sörensen, Klara Sondén, Anders Sönnerborg, Karin Stålberg, Kristoffer Strålin, Jonas Sundén-Cullberg, Christopher Sundling, Thanadol Sutantiwanichkul, Fernanda Costa Svedman, Mattias Svensson, Elisabet Svenungsson, Tadepally Lakshmikanth, Khue Hua Tran-Minh, Hasan Türkez, Christian Unge, Per Venge, Marie Wahren-Herlenius, Jakob Woessmann, Hong Yang, Umit Haluk Yeşilkaya, Meng Yuan, Mujdat Zeybel, Cheng Zhang, Wen Zhong, Martin Zwahlen, Kalle von Feilitzen, Peter Nilsson, Fredrik Edfors, and Mathias Uhlén. A human pan-disease blood atlas of the circulating proteome. *Science*, 390(6779), December 2025.

[16] Griffin Rodgers, Christopher Austin, James Anderson, Aaron Pawlyk, Christine Colvis, Ronald Margolis, and Jenna Baker. Glimmers in illuminating the druggable genome. *Nature Reviews Drug Discovery*, 17(5):301–302, January 2018.

[17] Christine M. Cutillo, Christopher P. Austin, and Stephen C. Groft. *A Global Approach to Rare Diseases Research and Orphan Products Development: The International Rare Diseases Research Consortium (IRDiRC)*, pages 349–369. Springer International Publishing, 2017.

[18] Joannella Morales, Shashikant Pujar, Jane E. Loveland, Alex Astashyn, Ruth Bennett, Andrew Berry, Eric Cox, Claire Davidson, Olga Ermolaeva, Catherine M. Farrell, Reham Fatima, Laurent Gil, Tamara Goldfarb, Jose M. Gonzalez, Diana Haddad, Matthew Hardy, Toby Hunt, John Jackson, Vinita S. Joardar, Michael Kay, Vamsi K. Kodali, Kelly M. McGarvey, Aoife McMahon, Jonathan M. Mudge, Daniel N. Murphy, Michael R. Murphy, Bhanu Rajput, Sanjida H. Rangwala, Lillian D. Riddick, Françoise Thibaud-Nissen, Glen Threadgold, Anjana R. Vatsan, Craig Wallin, David Webb, Paul Flicek, Ewan Birney, Kim D. Pruitt, Adam Frankish, Fiona Cunningham, and Terence D. Murphy. A joint ncbi and embl-ebi transcript set for clinical genomics and research. *Nature*, 604(7905):310–315, April 2022.

[19] Richard A. Gibbs, Jeffrey Rogers, Michael G. Katze, Roger Bumgarner, George M. Weinstock, Elaine R. Mardis, Karin A. Remington, Robert L. Strausberg, J. Craig Venter, Richard K. Wilson, Mark A. Batzer, Carlos D. Bustamante, Evan E. Eichler, Matthew W. Hahn, Ross C. Hardison, Kateryna D. Makova, Webb Miller, Aleksandar Milosavljevic, Robert E. Palermo, Adam Siepel, James M. Sikela, Tony Attaway, Stephanie Bell, Kelly E. Bernard, Christian J. Buhay, Mimi N. Chandrabose, Marvin Dao, Clay Davis, Kimberly D. Delehaunty, Yan Ding, Huyen H. Dinh, Shannon Dugan-Rocha, Lucinda A. Fulton, Ramatu Ayiesha Gabisi, Toni T. Garner, Jennifer Godfrey, Alicia C. Hawes, Judith Hernandez, Sandra Hines, Michael Holder, Jennifer Hume, Shalini N. Jhangiani, Vandita Joshi, Ziad Mohid Khan, Ewen F. Kirkness, Andrew Cree, R. Gerald Fowler, Sandra Lee,

Lora R. Lewis, Zhangwan Li, Yih-shin Liu, Stephanie M. Moore, Donna Muzny, Lynne V. Nazareth, Dinh Ngoc Ngo, Geoffrey O. Okwuonu, Grace Pai, David Parker, Heidie A. Paul, Cynthia Pfannkoch, Craig S. Pohl, Yu-Hui Rogers, San Juana Ruiz, Anikó Sabo, Jireh Santibanez, Brian W. Schneider, Scott M. Smith, Erica Sodergren, Amanda F. Svatek, Teresa R. Utterback, Selina Vattathil, Wesley Warren, Courtney Sherell White, Asif T. Chinwalla, Yucheng Feng, Aaron L. Halpern, LaDeana W. Hillier, Xiaoqiu Huang, Pat Minx, Joanne O. Nelson, Kymberlie H. Pepin, Xiang Qin, Granger G. Sutton, Eli Venter, Brian P. Walenz, John W. Wallis, Kim C. Worley, Shiaw-Pyng Yang, Steven M. Jones, Marco A. Marra, Mariano Rocchi, Jacqueline E. Schein, Robert Baertsch, Laura Clarke, Miklós Csurös, Jarret Glasscock, R. Alan Harris, Paul Havlak, Andrew R. Jackson, Huaiyang Jiang, Yue Liu, David N. Messina, Yufeng Shen, Henry Xing-Zhi Song, Todd Wylie, Lan Zhang, Ewan Birney, Kyudong Han, Miriam K. Konkel, Jungnam Lee, Arian F. A. Smit, Brygg Ullmer, Hui Wang, Jinchuan Xing, Richard Burhans, Ze Cheng, John E. Karro, Jian Ma, Brian Raney, Xinwei She, Michael J. Cox, Jeffery P. Demuth, Laura J. Dumas, Sang-Gook Han, Janet Hopkins, Anis Karimpour-Fard, Young H. Kim, Jonathan R. Pollack, Tomas Vinar, Charles Addo-Quaye, Jeremiah Degenhardt, Alexandra Denby, Melissa J. Hubisz, Amit Indap, Carolin Kosiol, Bruce T. Lahn, Heather A. Lawson, Alison Marklein, Rasmus Nielsen, Eric J. Vallender, Andrew G. Clark, Betsy Ferguson, Ryan D. Hernandez, Kashif Hirani, Hildegard Kehrer-Sawatzki, Jessica Kolb, Shobha Patil, Ling-Ling Pu, Yanru Ren, David Glenn Smith, David A. Wheeler, Ian Schenck, Edward V. Ball, Rui Chen, David N. Cooper, Belinda Giardine, Fan Hsu, W. James Kent, Arthur Lesk, David L. Nelson, William E. O'Brien, Kay Prüfer, Peter D. Stenson, James C. Wallace, Hui Ke, Xiao-Ming Liu, Peng Wang, Andy Peng Xiang, Fan Yang, Galt P. Barber, David Haussler, Donna Karolchik, Andy D. Kern, Robert M. Kuhn, Kayla E. Smith, and Ann S. Zwieg. Evolutionary and biomedical insights from the rhesus macaque genome. *Science*, 316(5822):222–234, April 2007.

[20] Z. Yang. Paml 4: Phylogenetic analysis by maximum likelihood. *Molecular Biology and Evolution*, 24(8):1586–1591, April 2007.

[21] Bo Li, Victor Ruotti, Ron M. Stewart, James A. Thomson, and Colin N. Dewey. Rna-seq gene expression estimation with read mapping uncertainty. *Bioinformatics*, 26(4):493–500, December 2009.

[22] GTEx Consortium. The gtex consortium atlas of genetic regulatory effects across human tissues. *Science*, 369(6509):1318–1330, 2020.

[23] Itai Yanai, Hila Benjamin, Michael Shmoish, Vered Chalifa-Caspi, Maxim Shklar, Ron Ophir, Arren Bar-Even, Shirley Horn-Saban, Marilyn Safran, Eytan Domany, Doron Lancet, and Orit Shmueli. Genome-wide midrange transcription profiles reveal expression level relationships in human tissue specification. *Bioinformatics*, 21(5):650–659, September 2004.

[24] Damian Szklarczyk, Katerina Nastou, Mikaela Koutrouli, Rebecca Kirsch, Farrokh Mehryary, Radja Hachilif, Dewei Hu, Matteo E Peluso, Qingyao Huang, Tao Fang, Nadezhda T Doncheva, Sampo Pyysalo, Peer Bork, Lars J Jensen, and Christian von Mering. The string database in 2025: protein networks with directionality of regulation. *Nucleic Acids Research*, 53(D1):D730–D737, November 2024.

[25] Lars Juhl Jensen, Jan Saric, and Peer Bork. Extraction of microarray gene expression data from literature using text mining. *Nature Biotechnology*, 27(10):921–929, 2009.

[26] Ana Rath, Annie Olry, Ferdinand Dhombres, Maja Miličić Brandt, Bruno Urbero, and Segolene Ayme. Representation of rare diseases in health information systems: The orphanet approach to serve a wide range of end users. *Human Mutation*, 33(5):803–808, April 2012.
